# Personalized Noninvasive Respiratory Support in the Perioperative Setting: State of the Art and Future Perspectives

**DOI:** 10.3390/jpm14010056

**Published:** 2023-12-30

**Authors:** Giovanni Misseri, Luciano Frassanito, Rachele Simonte, Tommaso Rosà, Domenico Luca Grieco, Alessandra Piersanti, Edoardo De Robertis, Cesare Gregoretti

**Affiliations:** 1 Fondazione Istituto “G. Giglio” Cefalù, 90015 Palermo, Italy; giovannimisseri1987@gmail.com (G.M.); c.gregoretti@gmail.com (C.G.); 2 Department of Emergency, Intensive Care Medicine and Anaesthesia, Fondazione Policlinico Universitario A. Gemelli IRCCS, 00165 Rome, Italy; luciano.frassanito@policlinicogemelli.it (L.F.); tommasoros22@gmail.com (T.R.); domenicoluca.grieco@policlinicogemelli.it (D.L.G.); alessandra.piersanti@policlinicogemelli.it (A.P.); 3 Department of Medicine and Surgery, University of Perugia, 06123 Perugia, Italy; edoardo.derobertis@unipg.it; 4 Istituto di Anestesiologia e Rianimazione, Università Cattolica del Sacro Cuore, 00165 Rome, Italy; 5 Department of Surgical, Oncological and Oral Science (Di.Chir.On.S.), University of Palermo, 90133 Palermo, Italy

**Keywords:** CPAP, HFNOT, ICU, mechanical ventilation, noninvasive respiratory support (NRS), noninvasive ventilation (NIV), perioperative care, postoperative pulmonary complications

## Abstract

**Background:** Noninvasive respiratory support (NRS), including high-flow nasal oxygen therapy (HFNOT), noninvasive ventilation (NIV) and continuous positive airway pressure (CPAP), are routinely used in the perioperative period. **Objectives**: This narrative review provides an overview on the perioperative use of NRS. Preoperative, intraoperative, and postoperative respiratory support is discussed, along with potential future areas of research. **Results**: During induction of anesthesia, in selected patients at high risk of difficult intubation, NIV is associated with improved gas exchange and reduced risk of postoperative respiratory complications. HFNOT demonstrated an improvement in oxygenation. Evidence on the intraoperative use of NRS is limited. Compared with conventional oxygenation, HFNOT is associated with a reduced risk of hypoxemia during procedural sedation, and recent data indicate a possible role for HFNOT for intraoperative apneic oxygenation in specific surgical contexts. After extubation, “preemptive” NIV and HFNOT in unselected cohorts do not affect clinical outcome. Postoperative “curative” NIV in high-risk patients and among those exhibiting signs of respiratory failure can reduce reintubation rate, especially after abdominal surgery. Data on postoperative “curative” HFNOT are limited. **Conclusions**: There is increasing evidence on the perioperative use of NRS. Use of NRS should be tailored based on the patient’s specific characteristics and type of surgery, aimed at a personalized cost-effective approach.

## 1. Introduction

Perioperative care, also known as perioperative medicine, is the practice of patient-centered, multidisciplinary, and integrated medical care from the preoperative phase to the postoperative recovery [1]. “Anesthesiomics” [2] embeds this holistic and personalized approach intended to provide high-quality care, minimize complications, and reduce requirement of postoperative intensive care unit (ICU) admission and hospital length of stay (LOS). 

Perioperative respiratory complications are a leading cause of morbidity and mortality. General anesthesia induces a loss of muscle tone and a cephalic displacement of the diaphragm, yielding reduction of functional residual capacity due to atelectasis and postoperative hypoxemia. These mechanisms lead to perioperative respiratory dysfunction and predispose to postoperative pulmonary complications (PPCs) [3,4]. These include postoperative hypoxemia, atelectasis, pneumonia, acute respiratory failure, and acute respiratory distress syndrome, which may prompt the use of mechanical ventilation [5]. PPCs are a prominent cause of prolonged hospitalization and mortality [6]. Several scores have been developed to predict the occurrence of PPCs [7,8], the emergence of which is related to surgical factors (intraoperative positional changes, diaphragmatic splinting, direct surgical injury to the lungs, pleura, diaphragm, chest wall, abdominal wall, or to the nerves supplying the respiratory muscles) and patient factors and comorbidities (advanced age, smoking, cardiovascular and respiratory comorbidities, obesity, pregnancy).

Current research is aimed at optimizing perioperative oxygenation and mechanical ventilation for specific cohorts of patients in different surgical subspecialties [9,10].

Thus, efforts have been made to adopt personalized strategies, with the aim to reduce perioperative respiratory complications [11] and improve postoperative patients’ outcome and recovery.

The incidence of acute respiratory failure after surgery is variable: the need for reintubation and invasive mechanical ventilation among unselected surgical patients is approximatively 5–10% but can be as high as 30–50% in high-risk patients undergoing abdominal and cardiac surgery [12,13]. 

Preserving perioperative respiratory function is a mainstay for ensuring safe anesthesia administration, success of surgical procedures, and uneventful recovery. The perioperative use of noninvasive respiratory support (NRS) may be associated with improved respiratory function and reduced PPCs. The term NRS is used in reference to noninvasive ventilation (NIV), continuous positive airway pressure (CPAP), and high-flow nasal oxygen therapy (HFNOT) [14,15]. 

**Figure 1:**
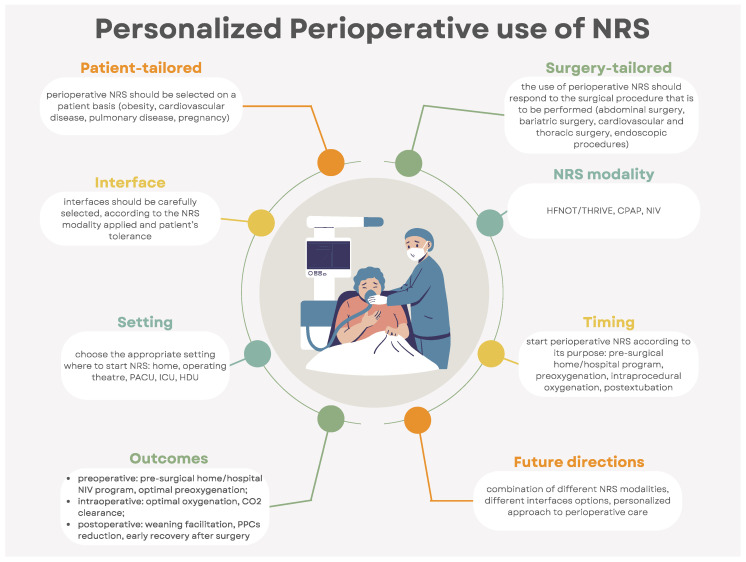
Infographic on the personalized perioperative use of noninvasive respiratory support (**CPAP**: continuous positive airway pressure; **CO**_2_: carbon dioxide; **HDU:** high-dependency care unit; **ICU:** intensive care unit; **NIV**: noninvasive ventilation; **NRS:** noninvasive respiratory support, **PACU:** postanesthesia care unit; **PPCs:** postoperative pulmonary complications; **THRIVE**: transnasal humidified rapid-insufflation ventilatory exchange).

**Figure 2:**
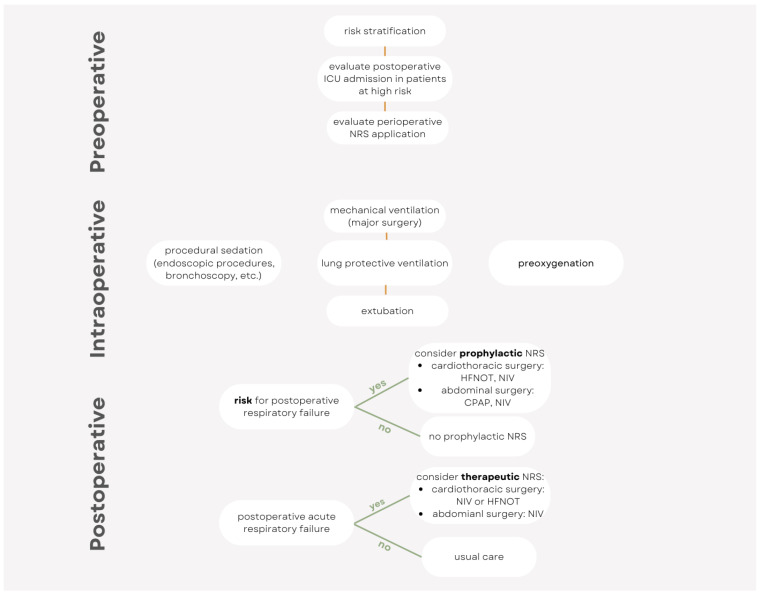
Simplified algorithm for the perioperative use of noninvasive respiratory support.

This narrative review’s aim is to give an overview (Figure 1) on the perioperative use of NRS across different surgical subspecialties and patient populations. Each of the three perioperative periods (preoperative, intraoperative, and postoperative) is discussed, highlighting the rationale and evidence (Table 1, Figure 2) on perioperative NRS use and the future areas of research in terms of personalized care.

## 2. Preoperative Use of Noninvasive Ventilation and CPAP

Preoxygenation before general anesthesia induction is a well-established procedure designed to increase oxygen reserves, in the attempt to delay oxyhemoglobin desaturation and prolong apnea time. The term apneic oxygenation refers to continuous oxygen flowing from upper airways to alveoli after anesthesia induction and during the apneic phase [31].

Preoxygenation with NIV limits alveolar collapse and atelectasis formation, which are responsible for hypoventilation, low perfusion–ventilation coupling and hypoxemia [31,32]. Through these mechanisms, NIV delays hypoxemia onset and lengthens the apneic period, with a prominent clinical effect in high-risk patients, such as those with difficult airways, patients undergoing bariatric surgeries, or emergency anesthesia for the critically ill [33].

Futier et al. [16] showed that the combination of NIV followed by early recruitment maneuvers can lead to an average increase in end-expiratory lung volume of approximately 700 mL when compared to conventional bag-mask preoxygenation. This increase in end-expiratory lung volume after NIV-based preoxygenation might be attributed to the recruitment of collapsed alveoli, consequently boosting oxygen reserves [34].

In the subgroup of obese patients, NIV significantly increases functional residual capacity, improves ventilation–perfusion ratio, and improves cardiac output [35]. When compared to spontaneous breathing, NIV can achieve a higher oxygenation within a shorter time frame [16] and increase duration of nonhypoxic apnea [36], making it particularly valuable in morbidly obese patients. Of note, a 5 min trial of CPAP during preoxygenation in obese patients has been shown to improve arterial partial pressure of oxygen (PaO_2_) after tracheal intubation compared to controls [17].

Preoperative CPAP may benefit patients with obstructive sleep apnea (OSA), a condition often undiagnosed before surgery and posing a substantial risk, including hypoxemia, hypercapnia, arrhythmias, myocardial ischemia, delirium, and unplanned ICU admissions [37]. Robust evidence for perioperative NIV use in this patient group is limited, but preoperative CPAP may control disease severity, reduce postoperative pulmonary dysfunction, and increase patient familiarity with the therapy [38].

Only few studies evaluated presurgical home/hospital NIV programs aiming at improving ventilatory status and familiarizing the patients with the NIV technique that will be applied after surgery. Perrin et al. [18] found that oxygenation and lung volumes were significantly better in patients using preoperative home NIV for 7 days before surgery than patients randomized to usual preoperative care. Of note, the incidence of major atelectasis was 14% in the NIV group and 39% in the control group. When considering cardiovascular and thoracic surgery, atelectasis are more commonly observed as postoperative complications, dramatically heightening the risk of respiratory distress. Prophylactic preoperative use of NIV has been proposed with the aim to reduce major PPCs after elective aortic surgery [39], demonstrating a reduction of early postoperative atelectasis and shortening ICU and hospital LOS.

The preOVNI study [40] investigated whether preoperative NIV could reduce PPCs after lung cancer surgery. In this randomized controlled trial (RCT), adult patients undergoing lung cancer resection were randomized to preoperative NIV (at least 7 days and 4 h/day) or conventional preoperative treatment. The authors concluded that there was no difference in PPCs between groups (42.6% in NIV group and 44.8% in no-NIV group). The rate of pneumonia was greater in the no-NIV group, but statistical significance was not achieved (28.0 vs. 37.7%, respectively; *p* = 0.08).

In conclusion, NIV represents a safe and effective tool for preoperative oxygenation, with a relevant clinical effect in patients prone to anesthesia-induced functional residual capacity loss and at high risk of difficult intubation with prolonged apnea following anesthesia induction.

## 3. Preoperative and Intraprocedural Use of High-Flow Nasal Oxygen Therapy

High-flow nasal oxygenation therapy (HFNOT) is an NRS technique that delivers high flows (60–80 L/min) of a heated, humidified, and blended air/oxygen mixture via specifically designed nasal cannulas [41].

A recent systematic review and meta-analysis on peri-intubation use of HFNOT revealed that apneic time and PaO_2_ measured after preoxygenation or after intubation were similar with HFNOT and conventional oxygen therapy (COT). In addition, the use of HFNOT also conferred no advantage in terms of peri-intubation hypoxemia, serious complications, or 28-day mortality [42]. However, it might be misleading to draw any conclusions for patients at higher risk of peri-intubation hypoxemia, as the mean apnea time in the selected studies was only <2 min.

In 2015, Patel et al. [19] coined the term transnasal humidified rapid-insufflation ventilatory exchange (THRIVE) to indicate the delivery of 100% oxygen at 70 L/min using the Optiflow THRIVE^TM^ apparatus (Fisher and Paykel Healthcare Ltd., Auckland, New Zealand) to adult patients with difficult airways undergoing otorhinolaryngologic surgery. The authors reported a median apnea time of 14 min and no episode of oxygen desaturation (SpO_2_ < 90%), proving that this approach represents an efficient way to mitigate the risk of hypoxemia following anesthesia induction and neuromuscular blockade [19]. Since then, a large body of evidence coming from studies conducted in the surgical population and during procedural sedation has elucidated the physiological mechanisms of this approach, as well as the important differences when the technique is applied to apneic or unparalyzed patients [43,44,45,46].

Provision of effective and extended oxygenation by HFNOT during rapid-sequence induction of anesthesia and during apnea has been examined in several settings [43,47,48,49,50,51], with some evidence also coming from morbidly obese patients, where HFNOT allowed prolonging of apnea time to 75 s compared to conventional facemask ventilation [52].

Tremey et al. [53] also described a “without the hands” technique for general anesthesia induction using HFNOT for preoxygenation and periprocedural oxygenation until tracheal intubation.

Hypercapnia is the most common complication with HFNOT and affects the overall apnea time. In an RCT including 118 patients undergoing laryngeal microsurgery under neuromuscular blockade, Min et al. [20] showed that, despite providing prolonged periods of apnea time, incidence of rescue interventions because of hypercapnia_,_ desaturations, and acidosis was more frequent for HFNOT compared to tracheal intubation. The pattern of carbon dioxide (CO_2_) rise (linear or nonlinear) as well as exact mechanisms regarding the extent of CO_2_ removal during HFNOT still need to be fully characterized. Some of the mechanisms involved were investigated in bench models: under apneic conditions, enhanced CO_2_ clearance may be explained by an interaction between entrained and highly turbulent supraglottic flow vortices created by HFNOT and cardiogenic oscillations that favor gas mixing [44,46].

Forsberg et al. [49] hypothesized that the increased difference in arterial and end-tidal CO_2_ in combination with high arterial oxygen levels, that were noted during apnea in patients undergoing laryngeal surgery and neuromuscular blockade during THRIVE [43], could be attributed to increased oxygen absorption and compression atelectasis, with subsequent shunt and loss of lung volume. However, lung volume changes assessed over time by electrical impedance tomography, albeit indicating an increased ventilation–perfusion mismatch, resulted in comparable reductions in lung volume during application of THRIVE and in mechanically ventilated patients. Comparable decreases in lung volumes estimated by electrical impedance tomography were also observed by Riedel et al. [21] when applying flow rates between 0.25 L/min and 70 L/min of humidified 100% oxygen in anesthetized and paralyzed adults, thus demonstrating that the induced increase in functional residual capacity occurring during spontaneous breathing [54] could not be replicated under general anesthesia [49]. Nevertheless, those conclusions cannot be prescinded from the consideration that respiratory mechanics differs significantly between paralyzed and unparalyzed states. With paralysis, dead space gas mixing and microventilation induced by pharyngeal pressure variations are inevitably altered [55]. However, a recent five-arm RCT [56] observed that differing flow rates of humidified 100% oxygen during apnea induced comparable arterial CO_2_ increases, thus questioning the existence of an additional ventilatory effect attributable to HFNOT [57].

Hypercapnia and desaturation are common concerns highlighted in most of the reported studies on procedural sedation. Other complications include gastric insufflation and airway ignition, the latter being theoretically possible in the case of concomitant use of lasers for surgical procedures. As a consequence, the need for cautious monitoring and a backup airway management plan, in particular when neuromuscular blockade is applied, is of primary importance [58].

Transcutaneous CO_2_ monitoring could be applied during procedural sedation. It is an accurate noninvasive alternative to arterial blood sampling, which provides clinically acceptable accuracy particularly when the sensor is applied to the earlobe [59].

The safety profile of HFNOT in procedural sedation, or whenever neuromuscular blockade is not needed, is less controversial [60]. A systematic review and meta-analysis including 15 RCTs (4451 patients) published in 2022 [22] showed with moderate-quality evidence that application of HFNOT compared to COT was associated with improved oxygenation, decreased need for airway intervention, and reduced procedure interruption in patients undergoing bronchoscopy and gastrointestinal endoscopic procedures. Those results were confirmed by another meta-analysis [61] evaluating the same outcome measures in 19 RCTs (4121 patients). Although 11 of the included studies coincided with those analyzed by Tao et al. [22], the latter included pediatric, cardiology, dental, and endovascular settings.

The effectiveness and safety of THRIVE as a stand-alone airway and breathing technique for airway management were recently evaluated during brief operative hysteroscopies under general anesthesia. In their study, Yuanyuan et al. [62] showed a reduced incidence of hypoxia, airway-related interventions, and involuntary movements during surgery in the THRIVE group when compared to COT, with high satisfaction scores both for anesthesiologists and gynecologists.

Frassanito et al. [63], in a pilot study including 20 patients undergoing operative hysteroscopies with the use of THRIVE apparatus, reported adequate gas exchange with acceptable short-term mean maximum transcutaneous CO_2_ levels of 51 ± 7 mmHg, no need for rescue airway interventions, and optimal comfort for the patients.

## 4. Postoperative Use of Noninvasive Ventilation (NIV)

Noninvasive ventilation (NIV) is widely used in the postoperative period to both treat (“therapeutic” or “curative” approach) and prevent (“preemptive” or “prophylactic” approach) postoperative acute respiratory failure [64]. There is a growing interest in its use in weaning strategies, bolstered by recent trials on early extubation practices (“facilitative” approach) [65].

The rationale behind its postoperative use resides on the improvement of alveolar ventilation due to reversal of atelectasis, which improves oxygenation and, partially, work of breathing. In addition, NIV does not require an artificial airway and it is achieved through interfaces which may be customized to improve adherence to treatment [66].

Current guidelines recommend NIV use for preventing postextubation respiratory failure in high-risk patients (moderate certainty of evidence), but not in low-risk patients (conditional recommendation, very low certainty of evidence) [67]. Regarding surgical patients, NIV finds a possible therapeutic application in the case of postextubation established respiratory failure (conditional recommendation, moderate certainty of evidence). However, there is no clear recommendation on the preventive use of NIV in the postoperative period.

The joint ESA/ESICM guidelines on NRS in the hypoxemic peri-operative/periprocedural patient [68] suggest using noninvasive positive pressure ventilation or CPAP immediately after extubation for hypoxemic patients at risk of developing acute respiratory failure after abdominal surgery (Grade 1B).

Patients requiring postoperative NIV should be admitted in predefined areas, such as postanesthesia care units (PACUs), ICUs, or high-dependency care units (HDUs), where a close monitoring of vital parameters, blood gas exchanges, and multidisciplinary evaluation can be performed [69,70] to avoid delayed intubation in case of deterioration of their condition.

### 4.1. NIV as Preventive Strategy in the Postoperative Period

The use of NRS after extubation in the postoperative period aims to mitigate the occurrence of acute respiratory failure and PPCs. So far, several risk scores have been validated to allow early identification of patients at higher risk for deterioration, thus preventing extubation failure [71]. Several trials have been performed with the purpose to analyze the effectiveness of preventive early NIV after major surgery, finding beneficial effects on PPC prevention [12,72].

Despite these findings, conflicting evidence emerges from larger randomized trials and meta-analysis. These inconsistencies are possibly explained by distinct target populations for whom postoperative NIV is used, suggesting that different modes of NRS may be successful when applied following specific types of surgery.

Zayed et al. [23] conducted a pairwise meta-analysis of nine RCTs on HFNOT, NIV, and COT after major surgery in patients at high risk for or with established postoperative acute respiratory failure. They found that neither HFNOT nor NIV influenced mortality rates. However, lower postoperative reintubation rates were observed with NIV or HFNOT over COT. Subgroup analysis showed lower reintubation rates with NIV after abdominal surgery (odds ratio (OR) 0.51 95% CI [0.26–0.87], whereas after cardiothoracic surgery reintubation rates were lower with both HFNO and NIV when compared to COT (OR 0.08 95% CI [0.03–0.21] and OR 0.08 95% CI [0.03–0.19], respectively).

The PRISM trial [73], a pragmatic clinical effectiveness trial investigating the real-world implementation of CPAP in PACUs, found that preventive CPAP started early after major open abdominal surgery does not reduce the incidence of postoperative pneumonia, reintubation rates, or death at 30 days. Therefore, its adoption as a preventive measure against PPCs could not be supported.

A recent systematic review and meta-analysis on the routine use of postoperative NRS after elective surgery [74], including 38 trials and 9782 patients, found that compared with standard care, the use of CPAP, NIV, or HFNOT did not reduce the incidence of pneumonia. In accordance with the previous findings, an open, multicenter RCT including patients at high risk for PPCs (Assess Respiratory Risk in Surgical Patients in Catalonia [7] (ARISCAT) score ≥ 45) showed that the preventive use of NIV has no beneficial effects over usual care in the incidence of postoperative acute respiratory failure. In this study, patients were randomly assigned to intermittent prophylactic face-mask NIV for 6–8 h per day or usual postoperative care, with the primary outcome being in-hospital acute respiratory failure within 7 days after surgery [75].

### 4.2. NIV as Therapeutic Strategy in the Postoperative Period

Therapeutic strategies are applied with the aim of avoiding reintubation and invasive mechanical ventilation in case of an already established postoperative acute respiratory failure. According to ERS/ATS 2017 clinical practice guidelines [67], NIV should be applied in these circumstances (conditional recommendation, moderate certainty of evidence) as a therapeutic strategy.

Several studies have shown that therapeutic NIV is able to improve clinical outcomes of patients undergoing major abdominal [12,76,77] and thoracic [24] surgery. Beneficial effects of postoperative therapeutic NIV have also been found in transplant procedures [78].

In their systematic review and meta-analysis, Faria et al. [72] found that compared to COT, CPAP or NIV may reduce the rate of tracheal intubation (risk ratio (RR) 0.25; 95% confidence interval (CI) 0.08 to 0.83; low-quality evidence). However, no differences were found for mortality (low-quality evidence) and hospital LOS. In addition, there was insufficient evidence to prove a potential link between postoperative NRS and complications such as anastomotic leakages, sepsis, and pneumonia-related sequalae. It is worth remembering that after abdominal surgery, the risk of gastric insufflation rises when pressure reaches values higher than 25 cmH_2_O. Hence, in upper-gastrointestinal surgical procedures, the risk of anastomotic leakage should be reduced by maintaining the pressure support level below 6–8 cmH_2_O [64].

Squadrone et al. [25] studied 209 consecutive patients who had undergone elective major abdominal surgery and developed postoperative hypoxemia. The patients were randomized to receive either COT or helmet CPAP in the recovery room. Patients receiving helmet CPAP had a lower reintubation rate (1% vs. 10%; *p* = 0.005) and a lower occurrence of pneumonia (2% vs. 10%, *p* = 0.02), infection (3% vs. 10%, *p* = 0.03), and sepsis (2% vs. 9%; *p* = 0.03) than patients treated with COT. When considering patients readmitted to an ICU with postoperative acute respiratory failure, NIV successfully prevented reintubation in 67% of cases.

In a multicenter RCT conducted among patients with acute respiratory failure after abdominal surgery, NIV use reduced the need for reintubation and for invasive mechanical ventilation and was associated with fewer healthcare-related infections when compared to COT [77].

### 4.3. Use of NIV after Specific Major Surgery Settings

The use of NIV after major cardiac and thoracic surgical procedures is a very attractive issue. Patients undergoing cardiac and thoracic surgeries are at higher risk of developing PPCs because of underlying pulmonary and cardiovascular dysfunctions. In these cases, preventive NIV may reduce PPC occurrence by reducing left ventricular preload and afterload, followed by an improvement of cardiac and respiratory functions [69]. Contradictory results emerge from studies conducted so far.

In their single randomized trial, Auriant et al. [24] found that NIV after lung resection decreased the frequency of intubation from 50.0% to 20.8% (*p* = 0.035) and mortality (13% vs. 38%; *p* = 0.045). In contrast with these findings, an RCT aiming to investigate whether prophylactic postoperative NIV could prevent acute respiratory postoperative conditions in COPD patients who had undergone lung resection surgery did not find any advantage on postoperative outcome. The authors found that acute respiratory failure rate was 18.8% in the prophylactic NIV group and 24.5% in controls (*p* = 0.20), reintubation rates were similar in the prophylactic NIV and control group (10/181 (5.5%) and 13/179 (7.2%), respectively, *p* = 0.53), and mortality rates were 5 and 2.2% in the control and prophylactic NIV groups, respectively (*p* = 0.16) [79].

The Bilevel Positive Airway Pressure Versus Optiflow study [26] was the first large, multicenter randomized controlled noninferiority trial comparing HFNOT and NIV in 830 patients developing hypoxemia during the spontaneous breathing trial or after extubation following cardiothoracic surgery. Treatment failure, defined as reintubation, switch to the other treatment, or premature discontinuation of the assigned treatment, was similar in both groups (21% for HFNOT and 21.9% for NIV, for the three strategies combined; absolute risk difference 0.9%, 95% CI −4.9% to 6.6%, *p* = 0.003), as were reintubations (14% in both groups). A post hoc analysis showed that there were no differences in the proportion of patients with treatment failure between HFNOT and NIV when these techniques were used as a weaning strategy or as a therapeutic strategy (27% for HFNOT vs. 28% for NIV, *p* = 0.93). When considering a preventive strategy for high-risk patients, treatment failure was lower in the HFNOT group than in the NIV group.

Interestingly, a recent network meta-analysis of 16 trials enrolling 3011 patients [80] found that NIV significantly reduced the incidence of PPCs when compared to standard of care (RR 0.67, 95% CI: 0.49 to 0.93; absolute risk reduction (ARR) 7.6%, 95% CI: 1.6–11.8%; low certainty) and the incidence of atelectasis (RR 0.65, 95% CI: 0.45 to 0.93; ARR 19.3%, 95% CI: 3.9–30.4%; moderate certainty); however, prophylactic NIV was not associated with a decreased reintubation rate or reduced short-term mortality. Further analysis showed that the highest ranked treatment for reducing the incidence of PPCs was NIV (83.0%), followed by HFNOT (62.5%), CPAP (44.3%), and standard of care treatment (10.2%).

Due to a restrictive syndrome and decreased chest wall compliance, obese patients are at higher risk of hypoxemia and PPCs. Increased body mass index is directly correlated with a loss of functional residual capacity by up to 50% of the preoperative values, with morbidly obese patients having significantly more atelectasis than nonobese patients, before induction, after tracheal extubation, and 24 h following laparoscopic surgery [81]. In addition, different comorbidities such as OSA, obesity hypoventilation syndrome, and cardiovascular and metabolic diseases are to be considered as predisposing factors for PPCs [12].

The obese patient, with or without OSA, might not respond to supplemental oxygen administration, being prone to postoperative oxygen desaturation and hypoxic episodes. Postoperative NIV could, therefore, mitigate the risk of extubation failure and the incidence of PPCs. However, evidence on the use of NIV after bariatric surgery is still limited.

A recent retrospective analysis evaluated the efficacy of short-term NIV use in reducing postextubation acute respiratory failure after biliointestinal bypass in obese patients in the PACU setting. The authors found an improvement of PaO_2_ (67.91 ± 3.03 vs. 60.14 ± 4.17, *p* < 0.001) and SpO_2_ (93.95 ± 2.09, *p* < 0.001) values in the NIV group, with usual postoperative care (oxygen therapy via a Venturi mask) being significantly associated with postoperative acute respiratory failure (RR 0.51, CI: 0.27 to 0.96, *p* < 0.05) [82].

Huerta et al. [83] adopted NIV in morbidly obese patients undergoing sleeve gastrectomy and open bariatric surgery, demonstrating that NIV increases PaO_2_ during the postoperative period (PaO_2_: 78.87 ± 8.31 in NIV group vs. 64.27 ± 6.33 in oxygen group). In addition, they found that only 15 out of more 1000 patients presented major anastomotic leaks, with only two cases related to postoperative NIV use.

The perioperative application of NIV in obese patients with positive airway pressure < 20 cmH_2_O is rarely a cause of NIV-related anastomotic leakage [84].

According to the meta-analysis conducted by Carron et al. [27], the postoperative use of NIV among obese patients was associated with a decreased risk of respiratory complications (RR 0.33, 95% CI: 0.16 to 0.66, *p* = 0.002) but not of reintubation after tracheal extubation (RR 0.41, 95% CI: 0.09 to 1.82, *p* = 0.3657) and unplanned ICU admission (RR 0.43, 95% CI: 0.16 to 1.15, *p* = 0.0937).

## 5. Postoperative Use of High-Flow Nasal Oxygen Therapy

The use of HFNOT has been widely studied and adopted after extubation of critically ill patients [85,86], particularly as a prophylactic measure to reduce the need for respiratory support escalation [68,87,88]. A series of RCTs evaluated the use of HFNOT with respect to COT [89] and NIV [90,91,92] in critically ill patients. Although initial trials demonstrated that HFNOT prevents reintubation in unselected cohorts of critically ill patients and performs as well as NIV in high-risk patients [93,94], recent data indicate that prophylactic HFNOT in all patients does not reduce the reintubation rate if patients treated with COT can receive a trial of rescue NRS (either HFNOT or NIV) before intubation [89,95]. In high-risk or obese patients, prophylactic NIV combined with HFNOT performs better than HFNOT alone [28,41]. This evidence indicates that HFNOT, even if not alternated with NIV, plays a crucial role in improving weaning outcome. Whether this evidence can be translated to patients undergoing general anesthesia has to be demonstrated by ongoing investigations.

The use of HFNOT has recently been proposed as part of an enhanced recovery program after major surgeries in patients at high risk for respiratory complications. This approach could improve functional recovery and reduce hospital LOS, with potential implications for reduced healthcare costs [96,97].

The effectiveness of preemptive HFNOT has been studied after cardiothoracic and abdominal surgery [26,98,99]. While data suggest that preemptive HFNOT can prevent respiratory support escalation in postcardiac surgery patients, with HFNOT being noninferior to NIV in hypoxemic patients [26], results in thoracic surgery may vary, likely due to differing control strategies or patient population [98,100]. In a randomized trial conducted after lung resection, patients receiving HFNOT showed a lower arterial CO_2_ and a reduced risk of postoperative hypercapnia than those treated with a Venturi mask [98]. In a meta-analysis by Chaudhuri et al. [29], prophylactic HFNOT reduced the rate of reintubation and respiratory support escalation when compared to COT in the immediate postoperative period after cardiothoracic surgery, an effect likely driven by patients at high risk and/or obesity. Positive pressure effect given by HFNOT application reverses atelectasis by increasing end-expiratory lung volume, finally reducing alveolar shunt fraction and enhancing arterial oxygenation [54,101,102]. Consistently with this, a recent randomized trial [30] involving patients undergoing major gynecological surgery in the Trendelenburg position showed that a preemptive two-hour HFNOT session following extubation improved postoperative oxygenation if compared to conventional Venturi mask oxygen therapy. This was primarily attributed to a reduction in atelectasis in the dorsal lung regions. Additionally, a quarter of patients in the HFNOT group received the assigned treatment with no supplemental oxygen, emphasizing that the beneficial effects of HFNOT were mainly due to high flow rather than supplemental oxygen provision [103,104]. However, HFNOT after extubation did not reduce the incidence of postoperative hypoxemia nor improved any other clinical outcome in patients at intermediate-to-high risk of PPCs [99]. The difference in outcomes between these studies may arise from population characteristics: patients undergoing laparoscopy in the Trendelenburg position had a higher incidence of postoperative hypoxemia compared to those of the OPERA trial [99], and patients undergoing lung resection may be at especially high risk of postoperative hypercapnia. Nevertheless, data from a recent meta-analysis did not support the routine use of postoperative CPAP, NIV, or HFNOT to prevent the occurrence of PPCs or pneumonia after surgery in adults [74].

Preemptive HFNOT may provide physiological benefits in specific patient subgroups but may not have a big impact in terms of patient-centered outcomes [99], especially when NIV use before reintubation is possible [89,95]. Therefore, preemptive postextubation HFNOT in surgical patients at low risk of PPCs necessitates a meticulous personalized assessment.

## 6. Future Perspectives: Artificial Intelligence and Machine Learning

Artificial intelligence and machine learning have the potential to personalize perioperative management and mechanical ventilation strategies for patients needing respiratory support [105]. Machine-learning models are complex mathematical constructs which could help the perioperative identification of patients at higher risk for deterioration while pointing out factors which could be optimized.

Optimization of perioperative respiratory support could be reached with the use of these models, and future research might focus on these issues in order to curb PPCs in high-risk patients undergoing major surgery. A recent study [106] validated a machine-learning model to predict and identify patients at high risk of NIV failure. The timely assessment of NIV efficacy and subsequent clinical decision are particularly crucial: if a patient is predicted to have a high risk of NIV failure, strict clinical monitoring could be provided and earlier reintubation considered, thus reducing mortality.

Several studies on critically ill patients have shown that machine-learning models may accurately predict extubation failure with remarkable accuracy [107] and identify patients in whom duration of mechanical ventilation might be longer [108]. However, there is a substantial paucity of data regarding the application of these tools in subjects undergoing general anesthesia. Therefore, development of new algorithms should be promoted to improve confidence and translate these approaches to everyday clinical practice.

Genomic data are increasingly applied in medicine, and the most difficult challenge to overcome is the huge amount of data generated and the difficulties related to translating these findings to clinical practice. With special reference to mechanical ventilation, there is no doubt that it has a direct impact on patients’ lung physiology. While ventilator-induced lung injury has been extensively explored, only a few experimental studies report that vigorous breathing effort during spontaneous ventilation can worsen lung injury and cause a phenomenon that has been termed patient self-inflicted lung injury [109]. The genetic determinants for these respiratory complications are unknown. However, the identification of novel therapeutic targets and the optimal NRS to apply is essential for progress in the understanding of these respiratory complications and for limiting their occurrence.

## 7. Conclusions

This narrative review revise much of the existing evidence on perioperative NRS use, reporting the most influential studies on the topic. What emerges is that personalized medicine is progressively shaping anesthesia practice and perioperative medicine, also for NRS. The evidence gathered so far brings profound implications for current clinical practice and should promote a careful reappraisal of guidelines on the perioperative use of NRS, especially when high-risk patients are considered.

Use of NRS should be tailored based on the patient’s specific characteristics and type of surgery, aiming at a personalized cost-effective approach.

## Figures and Tables

**Table 1:** Landmark studies on NRS use in the perioperative period (**ARF**: acute respiratory failure; **COPD**: chronic obstructive pulmonary disease; **COT**: conventional oxygen therapy; **ICU**: intensive care unit; **GA:** general anesthesia; **NRS**: noninvasive respiratory support; **HFNOT**: high-flow nasal oxygen therapy; **THRIVE**: transnasal humidified rapid-insufflation ventilatory exchange; **NIV**: noninvasive ventilation; **PACU**: postanesthesia care unit; **RM**: recruitment maneuver).

Study (Ref), Year	Design	Population Studied	Timing for NRS Application	NRS Modality	Findings
Futier et al. [16], 2011	Randomized controlled trial	66 morbidly obese 3 groups: (a) COT, (b) NIV, (c) NIV+RM after intubation	Preoxygenation before intubation	NIV	Preoxygenation with NIV+RM more effectively leads to an increase in end-expiratory lung volume with better oxygenation compared to COT
Coussa et al. [17], 2004	Prospective, randomized, single-blinded study	23 morbidly obese 2 groups: (a) CPAP = 10 cmH20 (b) CPAP= 0 cmH20	Oxygenation after induction	CPAP	CPAP groups had a significantly higher PaO_2_ after tracheal intubation
Perrin et al. [18], 2007	Prospective, randomized clinical trial	39 patients with preoperative FEV1 < 70% scheduled for elective lobectomy for cancer 2 groups: (a) without NIV (b) with NIV for 7 days at home before surgery and for 3 days postoperatively	Pre- and postoperative	NIV	NIV group had better oxygenation and lung volumes in the immediately postoperative period, with a better oxygenation until day 3 postoperative and lower hospital LOS
Patel et al. [19], 2015	Prospective, observational, cross-sectional	25 patients with anticipated difficult airways	Preoxygenation and continuing as postoxygenation during induction of anesthesia and neuromuscular blockade until a definitive airway was secured	THRIVE	With a median apnea time of 14 min, no episode of oxygen desaturation < 90% was recorded. THRIVE is an efficient way to mitigate the risk of hypoxemia following anesthesia induction and neuromuscular blockade
Min et al. [20], 2022	Prospective, randomized, parallel-group, noninferiority study	118 patients scheduled for laryngeal microsurgery 2 groups: (a) HFNOT group (b) tracheal intubation groups	During short apneic procedures (pre- and postinduction)	HFNOT	The frequency of rescue interventions due to hypercapnia, desaturations, and acidosis was higher for high-flow nasal oxygen therapy (HFNOT) compared to tracheal intubation. This led to the conclusion that HFNOT is not noninferior to tracheal intubation in terms of sustaining oxygenation
Riedel et al. [21], 2022	Single-center randomized controlled trial	125 adult patients scheduled for elective surgery 5 groups: (a) 0.25 L·min^−1^, endotracheal tube; (b) 2 L·min^−1^, continuous jaw thrust; (c) 10 L·min^−1^, continuous jaw thrust; (d) 70 L·min^−1^, continuous jaw thrust; and (e) 70 L·min^−1^, continuous laryngoscopy with a McGrath MAC video laryngoscope (Medtronic, Dublin, Ireland)	In anesthetized and paralyzed adults prior to intubation for 15 min of apnea	HFNOT	No differences between groups with respect to decrease in lung impedance or curve progression over the observation period
Tao et al. [22], 2022	Systematic review and meta-analysis	15 randomized controlled trials compared HFNOT with COT in patients undergoing endoscopic procedures	Nasal cannula during endoscopic procedures	HFNOT	The use of HFNOT was linked to enhanced oxygenation, decreased requirement for airway intervention, and reduced interruption of procedures in patients undergoing bronchoscopy and gastrointestinal endoscopic procedures (moderate-quality evidence)
Zayed et al. [23], 2020	Pairwise and network meta-analysis	9 RCTs representing 1865 patients at high risk for or with established postoperative respiratory failure comparing NIV, HFNOT, and COT	Postoperative time	NIV and CPAP vs. HFNOT	Neither HFNOT nor NIV had impact on mortality rates. But both NIV and HFNOT had lower postoperative reintubation rates compared to COT
Auriant et al. [24], 2001	Randomized prospective trial	24 patients with acute hypoxemic respiratory insufficiency after lung resection 2 groups: (a) NIV (b) no NIV	Postoperative time	NIV	The use of NIV in patients with acute hypoxemic respiratory insufficiency after lung resection can reduce the need for invasive endotracheal mechanical ventilation and reduce mortality rates
Squadrone et al. [25], 2005	Randomized controlled, unblinded study with concealed allocation	209 patients with severe hypoxemia after major elective abdominal surgery 2 groups: (a) oxygen (b) oxygen + CPAP	Postoperative time	Helmet CPAP	CPAP group had a lower intubation rate, occurrence rate of pneumonia, infection, and sepsis
Stephan et al. [26], 2015	Multicenter, randomized, noninferiority trial	830 patients undergoing cardiothoracic surgery with ARF in the postoperative period 2 groups: (a) HFNOT group (b) NIV group	Postoperative period for at least 4 h per day	HFNOT vs. NIV	HFNOT was not inferior to NIV. No significant differences found for ICU mortality
Carron et al. [27], 2016	Qualitative review and meta-analysis	29 articles on obese patients comparing perioperative NIV (all modalities) vs. COT	Just before and after induction of GA	NIV	NIV is associated with a significant improvement in oxygenation before tracheal intubation and benefits in oxygenation, clearance of CO_2_, and pulmonary function after induction. Postoperative NIV is associated with a decreased risk of respiratory complications, but not of reintubation and unplanned ICU admission
Thille et al. [28], 2021	Post hoc analysis of a randomized controlled trial	150 COPD patients 2 groups: (a) Patients treated with NIV alternating with HFNOT (b) Patients treated with HFNOT alone	Prophylactic NIV after extubation	NIV vs. HFNOT	NIV group had a lower reintubation rate. No differences in mortality in ICU
Chaudhuri et al. [29], 2020	Systematic review and meta-analysis	11 RCTs enrolling 2201 patients. 10 comparing HFNOT to COT. 1 comparing HFNOT to NIV	Preventing intubation in postoperative patients	HFNOT vs. NIV	Prophylactic HFNOT demonstrates a reduction in reintubation and the need for increased respiratory support when compared to COT during the immediate postoperative period after cardiothoracic surgery. This positive effect is likely attributed to patients at a high risk and/or those with obesity
Frassanito et al. [30], 2023	Single-center, open-label, randomized trial	83 patients undergoing major gynecological surgery 2 groups: (a) HFNOT (b) COT	Immediately after extubation	HFNOT	A 2 h session of HFNOT after extubation improved postoperative oxygen exchange and reduced atelectasis compared with a COT strategy

## Data Availability

Not applicable.

## Abbreviations

ARF	acute respiratory failure
ARISCAT (score)	Assess Respiratory Risk in Surgical Patients in Catalonia (score)
ARR	absolute risk reduction
CI	confidence interval
CO_2_	carbon dioxide
COPD	chronic obstructive pulmonary disease
COT	conventional oxygen therapy
CPAP	continuous positive airway pressure
GA	general anesthesia
HDU	high-dependency care unit
HFNOT	high-flow nasal oxygen therapy
ICU	intensive care unit
LOS	length of stay
NIV	noninvasive ventilation
NRS	noninvasive respiratory support
OR	odds ratio
OSA	obstructive sleep apnea
PACU	postanesthesia care unit
PaO_2_	arterial partial pressure of oxygen
PPCs	postoperative pulmonary complications
RCT	randomized controlled trial
RM	recruitment maneuver
RR	risk ratio
THRIVE	transnasal humidified rapid-insufflation ventilatory exchange
